# Ethics reflection groups in community health services: an evaluation study

**DOI:** 10.1186/s12910-015-0017-9

**Published:** 2015-04-17

**Authors:** Lillian Lillemoen, Reidar Pedersen

**Affiliations:** University of Oslo, P.O.Box 1130, Blindern, NO-0318, Oslo, Norway

**Keywords:** Ethics reflection, Community health services, Evaluation

## Abstract

**Background:**

Systematic ethics support in community health services in Norway is in the initial phase. There are few evaluation studies about the significance of ethics reflection on care. The aim of this study was to evaluate systematic ethics reflection in groups in community health (including nursing homes and residency), - from the perspectives of employees participating in the groups, the group facilitators and the service managers. The reflection groups were implemented as part of a research and development project.

**Methods:**

A mixed-methods design with qualitative focus group interviews, observations and written reports were used to evaluate. The study was conducted at two nursing homes, two home care districts and a residence for people with learning disabilities. Participants were employees, facilitators and service managers. The study was guided by ethical standard principles and was approved by the Norwegian Social Science Data Services.

**Results:**

We found support for ethics reflection as a valuable measure to strengthen clinical practice. New and improved solutions, more cooperation between employees, and improved collaboration with patients and their families are some of the results. No negative experiences were found. Instead, the ethics reflection based on experiences and challenges in the workplace, was described as a win-win situation. The evaluation also revealed what is needed to succeed and useful tips for further development of ethics support in community health services.

**Conclusions:**

Ethics reflection groups focusing on ethical challenges from the participants’ daily work were found to be significant for improved practice, collegial support and cooperation, personal and professional development among staff, facilitators and managers. Resources needed to succeed were managerial support, and anchoring ethics sessions in the routine of daily work.

## Background

There is extensive evidence that employees in health care services, both in hospitals, nursing homes, and home care services, frequently struggle with ethical challenges [1-8]. Although ethics support in hospitals is relatively well developed, both nationally and internationally [9-15], the development of ethics support seems to be sparser in community health services [1,5,16]. However, there is a need for ethics support in community health also [8]. Previous research indicates that ethics support in community health services should be closely tailored to the workplace, facilitated by a colleague [1] and concerned with the everyday ethical challenges employees experience [1,17]. However, there are few evaluation studies about the impact of ethics support in community health [18]. Recent research indicates that systematic ethics reflection is seen as a positive learning process among those participating [19]. Learning to reflect on the ethical challenges the employees face in their everyday work, and experiencing the benefits of it, such as relief of moral distress, are described as key reasons and motivations for implementing ethics reflection in clinical practice [20,21].

This paper presents the results from an evaluation study where ethics reflection in groups was implemented in community health services in a municipality in the central eastern part of Norway.

Norwegian municipalities differ with regard to size, population and organisation, but health services offered are generally public. Norwegians have a legal right to preventive health services, home-based nursing and institutional care services, e.g. nursing homes for the elderly and residential care homes for persons with learning disabilities, financed and provided by the state.

The community health services participating in this study are located in a municipality with approximately 120,000 inhabitants, where the publicly funded home-based nursing care is divided into six districts. The municipality’s 16 nursing homes and 8 residential care homes are also publicly funded. While staff in home-based nursing care and nursing homes, mainly are nurses, auxiliary nurses and nurse’s aides, staff in the residential care homes are mainly social educators and care workers.

### Aims and context of the study

The aim of this research was to explore how ethics reflection in colleague groups was experienced and evaluated by the employees, facilitators, and service managers. The study was part of a larger research and development project carried out as collaboration between five health care services in the community (two nursing homes, two home-based nursing care service districts and one residential care home for persons with learning disabilities), the community health management, and the Centre for Medical Ethics (CME) at the University of Oslo. CME was responsible for the research and evaluation part of the project, and participated also as expert consultants and teachers in other parts of the project. The project’s action-oriented approach meant that the services had a major influence on the design of the project phases. The authors - researchers in clinical ethics at CME - along with eight resource persons from the five community health services played key roles throughout the project. Five of the resource persons from the community health care services - four nurses and one social educator - had their daily work in the community health care services. In addition, they were all trained as facilitators of ethics reflection in their departments. The other three resource persons from the community health services were responsible for quality improvement work in the health care services, and one of them served as the leader of the larger research and development project.

The project was carried out in the period 2008 – 2011, and consisted of three phases. Phase one; a survey and group interviews focusing on: a) The ethical challenges employees struggle with, and the extent of these challenges. b) Employees’ need for ethics support. Findings from the survey have already been published [1]. Phase two; implementation of ethics support based on the survey and interviews. Several measures were identified as needed, and implemented. Among these were: a four hour ethics course for all employees, and establishing meeting places on wards where the staff collectively (in interdisciplinary groups) could reflect on ethical challenges in their everyday work. A brief description of the ethics reflection groups is given in the text below. This phase also included education and training of staff members to facilitate the ethical reflection on wards. In order to make course correction the project was evaluated every six months. Phase three; two years of systematic ethics reflection groups that included all employees. Despite being invited, the doctors did not participate. The evaluation study focused on phase two and three. A more thorough description of the entire project’s schedule and content have previously been published [22]. The research questions we wanted to answer in the evaluation study were:

1. What ethical challenges have been discussed?
2. What is the significance of ethics reflection groups on health care professionals’ practice?
3. What have been the success factors and barriers to participation in reflection groups?

### Ethics reflection groups

The organisation of ethics reflection groups relied on close collaboration between the ward leader and the facilitator. Ethics reflection groups took place at scheduled times, 1 to 1 ½ hour weekly, or every second week. The groups were open without fixed members; and those who were at work had the opportunity to participate. The health care professionals sat down together and collectively reflected on ethical challenges from their daily practice. Those participating brought cases to reflect upon, and the group chose which case to discuss. At one location they used constructed cases, based on current challenges on the ward. The reflection was carried out systematically, led by the facilitator and structured by the CME Method:

1. What is the ethical problem?
2. What are the facts of the case?
3. Who is involved, and what are their views?
4. Which values, laws and guidelines are relevant?
5. What are the alternative courses of action?
6. Overall assessment.

In addition to the CME Method, the groups developed rules for the activity, such as:

- We will listen to each other.
- We will not judge each other.
- We will strive to understand each other.
- We help each other to respect confidentiality.

## Methods

We conducted three focus group interviews at the end of phase three. The participants were managers or other employees (facilitators and participants in ethics reflection groups on wards) of health care services in this ethics project. The project leader, employee of the municipality, was responsible for the recruitment, and gave potential participants oral and written information about the evaluation project.

The first interview took place in a group with seven employees - nurses and auxiliary- and nurse’s aides - who had participated in ethics reflection groups at their workplace. With the exception of one of the two participating nursing homes, all workplaces participating in the project were represented in the interview. The second interview had five ethics facilitators, all members of the resource team, and representing all workplaces participating in the project. The final interview was conducted with five service managers representing the five participating health care departments. Different levels of expertise or power may hinder the participants in focus groups from expressing what they think or feel [23]. The reason for dividing the participants into three distinct groups for different kinds of stakeholders was that we wanted to create an environment where the participants felt comfortable to share their experiences and insights, even negative, experiences that they could be more reluctant to report if e.g. managers were present. We prepared three different interview guides, and the questions posed to each group differed slightly, to elicit answers to the research questions from the participants various perspectives, experiences and responsibilities. For example, when staff and facilitators where mainly asked about their experience of possible changes in practice related to the ethics reflection groups, managers where mainly asked about the signals they had received, what they had been told and possibly observed of changes in practice.

Each interview took about two hours and was audiotaped and transcribed verbatim. We were two interviewers (the authors); one primarily responsible for interviewing, the other responsible for the technical equipment and follow-up questions. The reason for using focus group interviews was that it can facilitate an exchange of experiences and views among participants (when group members hear the ideas of others, they are more able to identify other things that could potentially help or not); we can include several participants in each interview, and thus generate richer data. We acknowledged the risk of participants agreeing with others in the group as a potential problem, and encouraged the participants to express their opinion, especially if it differed from what others in the group expressed.

For the interviews we used a thematically organised interview guide with three main topics: 1) The implementation of ethics reflection in colleague groups (frequency, use of time, participation, success factors and barriers for participation). 2) The ethics reflection (challenges discussed in the groups, methods, discussions among group members). 3) Results or significance (e.g. for the challenges discussed, attention to ethics in their everyday work, for the ability to deal with ethical challenges, for the quality of health care services, for the patients and their families, for the work environment).

In addition to the interviews, we also had written documentation from the facilitators, notes from the reflection groups and bi-annual evaluation. The notes contained descriptions of how ethics reflection groups were implemented in the department, ethically challenging situations discussed, and the number of staff that had participated in the ethics reflection groups. We also asked the facilitators to document the significance they considered the reflection to have, for example what it meant for the staff’s awareness of ethics, and their ability to deal with ethical challenges. The notes also contained reflections upon the impact this initiative had on quality of health care. Furthermore, we took field notes during observation (e.g. as co-facilitators), training sessions, supervision, project meetings, and other experiences we as researchers have gained through working with the larger project, and with the facilitators and the reflection groups in particular.

### Data analysis

The tape-recorded data was transcribed and then analysed. We conducted a qualitative content analysis of the transcriptions and written notes, searching for answers to our three research questions (see above). Both researchers read the written material, and analysed the various parts of the texts together. Then the first author had the primary responsibility for more detailed analysis and condensation. A basic issue when performing qualitative content analysis is to decide whether the analysis should focus on manifest or latent content [24]. The text’s manifest content is the most visible or salient components, while the text’s latent content involves an interpretation of the underlying meaning of the text [25,26]. The focus of our analysis has been the manifest content and the categorisation has been purposive in the way that it has focused on the participants’ expressions answering our three research questions mentioned above. After agreeing on the main themes and subthemes, we categorised and condensed the relevant content of the text.

### Ethical considerations

When designing the evaluation, we were guided by ethical standard principles [27]. All participants (staff members, facilitators and managers) were informed verbally and in writing about the evaluation and that it was voluntary to participate. They gave their written consent to participate before the interviews started. When referring to situations in the interviews that had been discussed in the reflection groups, we reminded the participants to remove person-identifying information.

According to the Norwegian Act on medical and health research (ACT 2008-06-20 no. 44, § 2, § 4 and § 9) ethical review by the Regional Committee for Medical and Health Research Ethics (REC) is only required when the study is defined as medical or health research [28]. REC, which was consulted in the planning of our study, defined this study to be outside the legal definition of medical and health research in Norway. Thus, necessary ethical approval was instead given by the Norwegian Social Science Data Service (NSD) after reviewing our study. NSD is the Data Protection Official for all Norwegian universities [29].

## Results

Starting out with our three questions, we identified seven themes. In the following we will focus our presentation on the analysed and thematised findings which answer the research questions. Findings answering the research question that asks what ethical challenges that have been discussed are analysed and thematised as: “Patient autonomy” and “Family participation”. These themes will briefly be summarised in the beginning. Findings answering the question of the significance of ethics reflection groups are analysed and thematised as: “Improved quality”; “Collegial support, team cooperation, and mutual learning” and “Professional and personal development”. Findings that answer the question of success factors and barriers are analysed and thematised as: “Plan, structure and anchoring” and “Cases from the staff’s workday”. The opinions that emerged in the three interviews often coincided. Where differences appeared, we have made it explicit in our presentation below.

### Ethical challenges that have been discussed

The most frequent discussions in the ethics reflection groups have been ethically challenging patient cases from the participant’s workday. *“We have had cases about our patients throughout, about problems in our workday – simply like that - ethical issues”* (F3). The focus of these discussions has often been on patient autonomy and the staff’s struggle to identify alternative courses of action. This has been particularly prominent in situations where employees have been responsible for the care of patients over many years and old habit plays a significant role in the nursing care. In Table 1 some specific examples of ethically challenging situations are presented. These examples concern mostly patient autonomy, a common repetitive problem.

**Table 1:** **Examples of ethical challenges that have been discussed**

**a) Patient autonomy**	**b) Family participation**
An alcoholic patient who isn’t able to go out and do his shopping. Family members are buying alcohol for him. The nurse brings the situation to the reflection group and talks about the problem his drinking causes and asks if they can deny him alcohol.	A son expresses strong dissatisfaction with his father’s care and treatment. He threatens to bring a lawyer. The nurse brings the situation to the reflection group and feels compelled to do as the son demands, even though she disagrees.
An overweight woman, living in her home, spending every hour, every day, every week on the sofa. She sits and lies in urine and feces that get stuck to her skin and she refuses to allow them to help her freshen up. The nurses assess the situation as terrible and bring the situation to the reflection group – what can we do? Use force to care for her?	An elderly woman, which we considered to be dying, with family members that demanded that we give her medication and IV fluid treatment. We believed that the treatment would aggravate the old lady’s distress and increase her suffering. But because the family was steadfast and demanded treatment, we did as they asked.
A resident with learning disability who is very overweight and loves food. In the reflection group the nurse reflects on whether it is justifiable to deny her more food when she has eaten her portion and asks for more?	A daughter is very upset because her father, who is a patient at the nursing home, is in a relationship with one of the other patients. She accuses the employees of not having prevented the relationship, and demands that they bring it to an end. Is it right to hinder the couple in being together? The relationship between them means a lot for their joy and satisfaction.

Another main group of ethical challenges discussed in the reflection groups focus on family participation. Family members’ dissatisfaction with the care patients receive, or with unacceptable or improper behaviour by employees. These discussions were often about how employees ought to, or could have handled the ethically challenging situations. Should the staff act as the family demands, in accordance with the patient’s wishes, or according to their own professional judgments? Another aspect of the situations that often were discussed was whether the family’s complaints were reasonable, as well as how to improve the conditions.

### The significance of ethics reflection groups

All three interview groups emphasise the positive significance ethics reflection groups have had on the climate of cooperation, not simply among the staff, but also with patients and their families. Ethics reflection groups are perceived as having a positive significance on employees’ attention to ethics, and to their ability to deal with ethical challenges they face in their everyday work. Now they do not struggle alone with the challenges, but bring them to the reflection session to discuss them with their colleagues.

Some of the managers and facilitators in the group interviews expressed confidence that the systematic ethics reflection would lead to reduced absenteeism, and increased interest among nurses to work in community health facilities, such as nursing homes. In the group interview with the staff, the participants were more doubtful whether this would happen.

#### Improved quality

Prior to participating in ethics reflection groups, the employees’ actions and considerations were often characterised by old habits. Two years of ethics reflection groups have changed their attitude. Today they critically question practices and conditions they previously took for granted. They see things in a new way and find new solutions. This has led to important changes in practice.

An indicator of improved quality, are the staff’s and the facilitators’ descriptions of the positive consequences of the ethics reflection for the patients; *“We have residents who have lived here for so many years that we no longer questioned our choices. Because of the discussion in the reflection group there have been changes. Some might consider them small, but for the resident it means a lot”* (F5). See an example below: “Reflections on a cup of morning coffee”.

### Reflections on a cup of morning coffee

In one of our ethics reflection groups, one of the staff members questions the practice we have followed for years for one of our residents. What is the reason for refusing the resident her desired cup of coffee in the morning before she does anything else? This is something she repeatedly asks for, often rather loudly. There are quite a few of us who need a cup of coffee to wake up in the morning.

In the ethics reflection group we discover that the resident has fixed procedures as part of an effort to develop ADL skills, and this is part of her routine; no coffee before morning grooming and tooth brushing is complete. Through the discussion we realise that this is a practice that can be traced 20 – 30 years back in time, to the time she lived in a central institution. Reflecting on the situation, the staff member questions why this is still practiced. Hasn’t she learned these skills well enough through all these years? How can we justify refusing her the coffee she so strongly wants?

The result of the ethics reflection group this day was a satisfied resident. Today we offer her a cup of coffee when she wakes up, and sitting on the sofa watching morning-TV she enjoys the coffee before she completes her morning routines.

The management is also convinced that ethics reflection benefits the patients and their families, and they have seen that the initiative have led to increased knowledge about ethics among the employees. Now the managers find that they are challenged by the employees. Referring to professionalism and ethics, the staff challenges the managers to give them alternative courses of action; *“The staff now set higher standards for themselves and their own service, so that’s good,”* (M5) one of the managers says, indicating that it is more challenging in a positive way, for the managers to face critical questions.

Both the staff and facilitators agree that having ethics reflection in the workplace has contributed to a better relationship with patients and their families; “*I think it does something to our attitudes towards patients and their families. They notice it. It leads to better quality and attentive service. It raises awareness”* (M3). One positive outcome is that patients’ and relatives’ participation has been strengthened, and the change is attributed to the staff now being more conscious of it; “*The residents are more seen and heard now. There is more user participation”* (S3). The staff talk more about the importance of being aware of the patients’ facial expressions so that they do not act against the patients’ will, and they find that they use coercion less frequently; *“We have many residents without the ability to speak and therefore it is important to hear, see, and interpret them. There is more focus on this now; we talk about it – There’s more user participation”* (F3).

Staff awareness of their own practice has resulted in improved practice and more confidence in the performance of the service, a change that is solely positive; “*When you’re more confident, then you will be more motivated. Increased professionalism and increased focus on the resident,”* (M2) as one of the managers expresses herself. The participating managers consider the choice they made, focusing on ethics reflection, to be worthwhile.

### Collegial support, team cooperation, and mutual learning

Ethics reflection is perceived as having had a positive impact on the relationship between colleagues. Learning together, for instance about laws and regulations, which they prior to the project had little knowledge of, strengthens their relationship. The staff comes from many different countries and cultures. This was previously perceived as a problem. Now it is described as an opportunity for learning. Time and space to sit down and talk together in a busy workday makes it possible to discover new aspects of each other. They became aware that some colleagues know things they don’t know, and they learn together and from each other; “*I think we grow in our professional environment, that we become familiar with laws and regulations. It’s not every day we can sit down and read, but sometimes we have colleagues who are good with laws and that is very useful”* (F3).

In addition, the collegial support they have developed in the reflection groups is a good contribution to strengthening the relationship between staff members. They discuss challenging situations together, and the cooperation and support they give each other reduces the level of conflict; “*Now there is less gossiping in the halls”* (F1). The cooperation between employees has led to increased motivation to do a good job; *“I think everyone feels that talking together about the dilemmas makes the workday easier. You can share your struggles with colleagues. These groups make it less ‘scary’ to talk about the challenging situations”* (S2).

Both facilitators and managers emphasise fellowship as a positive result of ethics reflection. Auxiliary- and unskilled staff also find that their opinions matter more; “*What I think doesn’t matter, so I say nothing. ´I think I heard such things more often before”* (F1). The formal and specified arena for ethics reflection, has in turn resulted in ethics reflection elsewhere, in formal and informal meeting places; “*For example in staff meetings, - and now we discuss ethics not only on the established arena”* (M3).

The managers, as well as employees and facilitators, confirm that ethical reflection is positive for collegial solidarity and cooperation. Dialogue is strengthened, there is more openness for sharing experiences of ethical challenges, and there is more tolerance between employees*.* One of the managers points out that it has been a turnaround to work systematically with ethical challenges in the unit. Prior to this the staff struggled alone with the dilemmas. Now they work with their colleagues to find out how to deal with ethically challenging situations; “*We often work alone and have no one to discuss with, and then we have the duty of confidentiality. Having the ethics group is very good; we can discuss how to deal with things [in the groups]. Most of us have moments of discovery – gosh – why haven’t I thought of that?”* (S2).

Lack of time, which was a frequently used apology for not prioritising the reflection groups, is not used as much anymore. One of the managers talks about a drawing she has shared with the staff to make them prioritise participating in the groups; “*It is a drawing that shows a person pulling a cart with a square wheel. Another person runs to help, bringing a round wheel. Then the person pulling the cart with the square wheel says, - no I do not have time”* (M5).

### Professional and personal development

The staff describes the consequences of ethics reflection as a process of change, a professional and personal development. When talking about the learning process, some conditions are particularly highlighted by the staff. They feel more confident about themselves and the work they do; “*You grow personally when you are involved in this, and you become more confident”* (S3). The fact that they can discuss issues with colleagues, get confirmation that these are difficult questions, and sometimes get input about how the situation can be handled, has reduced the feeling of being alone with the responsibility; *“You mention things in order to hear what other people think about it, if they think the same way or differently”* (S1). Ethics reflection has led to changes in their everyday work. When they face ethically challenging situations now, they are more confident that they can manage to solve the problems they face. Staff, as well as facilitators, points to the CME reflection model as important for the learning process. They find the model to be a help to reflect on, and weigh different arguments in favour of and against the alternative actions available. These results are confirmed in some of the field notes from participant observations in ethics reflection groups.

The staff, facilitators, and management all highlight increased ethical and critical awareness as a consequence of the reflection; “*I notice in myself that I have been ethically conscious in my daily work. Day and night, I have ethics in my head and it was not like that before”* (S3). When asked how they understand the concept of ethics, the employees reply that ethics, for them, is reflection on practice. The facilitators explain the necessity of scheduled ethics reflection since there is too little time and space for reflection during a hectic work day; *“You do not reflect on everything you do because you do it every day, and you do not think about what’s in it for the patients. You are on autopilot; do not think about what is actually going on”* (S3).

### Success factors and barriers

The interviews uncovered differences between the various departments when it comes to organisation and the staff’s attendance in the ethics reflection groups. Four out of five departments describe ethics reflection groups as a well-established practice, and there is great participation from the staff. In one department they consider ethics reflection groups to still be in an initial phase, a fragile established practice. During the project period, this department has been characterised by high turnover and management changes. Yet, even here, where they are struggling with implementation, they find great commitment among the staff when a reflection session actually takes place.

### Plan, structure, and anchoring

All participants in the interviews, employees, facilitators and managers, point out how important it is that ethics reflection is part of the ward’s action plan. If ethics reflection is a part of the action plan, this in turn leads to an assessment of whether or not the reflection is carried out as planned, i.e. a formal commitment and some sort of evaluation (often informal).

Based on feedback from the staff, the managers’ main conclusion is that the project’s specific focus on ethics and reflection has been important. All participants consider scheduled times for ethics reflection groups to be essential. That ethics reflection groups were given priority by the managers, and resources were allocated to it, were considered to be necessary success factors.

Facilitators, as well as participants in ethics reflection, find the reflection method (the CME method) to be an important success factor. This reflection method helps them structure the discussion, and stick to the case; “*That is why the method is so useful; it helps you to keep some structure. It’s not just chatter; that nothing comes out of…”* (F3).

The facilitator’s have taken anonymised notes from the discussions, using the CME method to structure also the notes. The notes are put in a binder available to the staff. The notes enable employees who have not been able to participate in the reflection, to read what has been discussed. *“We call it the ward’s ethics folder, every ward should have one”* (F1). Thus, all employees at the ward can see what was discussed and the main content of the discussion.

The facilitators emphasise the importance of anchoring ethics reflection throughout in the health care services, not only by the top management. They consider it to be even more important that ethics reflection is anchored by the ward manager. Both implementation and employee participation in ethics reflection needs to be explicitly requested by the manager. Anchoring among employees is also described as an important factor for success. In particular, the staff’s experience of ethics reflection groups as a meaningful use of time is a necessity in a busy workday. I.e. they must experience the measure as important and useful in order to prioritise it.

### Cases from the staff’s workday

The majority of the facilitators invite the employees to bring ethically challenging situations from their own workday into the reflection group. One of the facilitators constructs cases which resemble what the staff face in their workday; “*We often have ethically challenging situations in our unit. When it happens, I make a case of it. Often the result is that we chuckle and laugh because we see ourselves: ‘Oh My God this is so stupid. That’s the way it is, and it should not be that way’”* (F1). Whether using constructed or experienced challenges, they all found it successful to have specific cases that are relevant to their everyday practice to reflect upon. One advantage of using constructed cases is that possible issues of confidentiality are more easily are avoided.

However, that ethics reflection is focused on the concrete and the practice-oriented, and the employees’ needs and challenges, makes the group discussion particularly valuable; *“They [the employees] reflect over the things happening on the wards. It is not general cases that do not mean very much, but they are going into specific situations that they are involved in. They find that they benefit from it, for example in relation to particularly challenging patients that we have on the ward”* (M2). If the discussion of real cases generates new and better solutions, the solutions are likely to be used afterwards. Thus, the usefulness of the reflection groups becomes more obvious.

## Discussion

The aim of this study was to examine how managers, facilitators and participants in ethics reflection groups, experienced and evaluated these groups. First of all, it is obvious that some of the ethically challenging situations that have been discussed in the reflection groups will probably not always, and perhaps especially not by ethicists, be perceived as ethical problems, but more as problem of professional, technical, organisational, and collegial character. Nevertheless, these are situations that staff members, facilitators and managers, experience and present as ethical problems in their everyday practice.

Like other recent studies from community health care, our study shows that ethics reflection in groups, above all, is experienced as a very positive and important measure [19-21]. The participants describe ethics reflection as a process that makes them more aware of what patients and relatives express, of the patient’s needs, but above all, more concerned with the patient’s right to participate in decision-making processes.

The participants, nurses and auxiliary nurses, understand ethics as a critical reflection on their own clinical practice, and find that this process leads to an increased ethical awareness. Ethical awareness is an important part of health care workers’ clinical competence, and the measure must therefore be considered to be professionally stimulating and developing. The way the participants describe their new practices; e.g. strengthened ability to communicate with patients and relatives, sharpened awareness of patients’ wishes, feelings and well-being, finding new and better solutions, team cooperation and mutual learning, are important aspects of professionalism and good practice. Ethics support in the form of systematic ethics reflection in groups also seems to affect the employees’ practice performance, and their ability to reflect on and criticise their own practice in a constructive way. This is strongly related to what the participants describe as a success factor: that ethics reflection is based on the employees’ practice experiences. It is the employee’s actual and perceived challenges they investigate; reflect on and discuss. Not theoretical or potential situations or general values, but real, challenging situations that the professionals need help looking at, and trying to find new ways to cope with in respectful dialogue.

This study indicates that the systematic and respectful dialogue between employees, and the reflection on ethical problems in their own workday, can be one important way to new and better solutions and thus improved practice. Using a simple and systematic method of reflection, facilitating adequate training, and organisational backing seem to be key success factors. These findings also resemble findings from evaluation studies of clinical ethics support services in hospitals [30,31].

Assuming that our very positive findings are valid, how can we understand or explain this? First, it is obvious to us that ethics reflection groups appear to meet a need for professional development that otherwise may not have good growing conditions. Increasing demands for efficiency, target management and evidence seems prominent, while dialogue, reflection, professionalism and relationships have poorer conditions in modern health care. Second, it seems that being invited to reflect on clinical practice leads to a good learning process. According to Argyris and Schön, learning occur both as single- and double loop [32,33]. Which learning it is that has taken place depends on the outcome and process. Doing more of the same, perhaps using new techniques, greater certainty and quicker performance, charactarises single loop learning. While double loop learning requires a qualitative change, a new stage characterised by qualitatively new content, and the result is professional development [34].

The participants’ description of the significance of ethics reflection indicates double loop learning and professional development. How can this be explained? The working day without ethics reflection provides little opportunity for so-called double loop learning; this may partially explain why ethics reflection is considered “profitable” and significant. Another interesting fact is that the employees seem to define ethics in accordance with double loop learning when they explain ethics as reflection on practice. Unlike the automated actions that characterised the participants’ previous practice, they have discovered new aspects of their own practice, they ask questions they did not ask before, and they have become aware of new ways to deal with ethical challenges. Participation in ethics reflection groups seems to have had a positive impact on the staff’s professional development. Community health care is often characterised by time constraints and the inability to meet all the patients’ needs, with little time for reflection on clinical practice. The possibility for nursing staff to sit down and reflect together can be one explanation for the overwhelmingly positive response.

Instead of thinking that this takes time, the managers consider ethics reflection to be a valuable investment, a win-win situation. Not only has the measure led to increased knowledge and competence in ethics among the staff, and thus improved quality of service, the managers also consider the measure to be an important recruitment initiative.

The employees describe ethics reflection and competence to be a necessary part of their work. Although the majority has received some ethics training in their education, the employees consider ethics reflection in the workplace to be further training of great importance. A measure like ethics reflection compensates, to some extent, for the wide variation there is in the employees’ ethics education. Furthermore, it seems to improve the interdisciplinary learning environment. The differences between the employees, both in cultural background and education, no longer appear to be a threat, but a source of learning.

### Some lessons learned

One important lesson learned is to base the ethics reflection on cases from the participants´ own practice right from the start. To discuss general terms, concepts, ethical values and principles are perceived as less important and fruitful.

An interest in ethics, as well as seeing ethics reflection as important, is not sufficient selection criteria for ethics facilitators. Participating in and observing the facilitators in the training program, as well as their reflections and discussions in network meetings, revealed how demanding some of the facilitators found their role. We learned that knowledge and skills are important criteria, criteria it is important not to under-communicate when facilitators are selected. Implementing ethics reflection led by colleagues as facilitators therefore requires an education program that includes exercises to develop necessary skills. The exercises should be integrated throughout the education program, from the very beginning. One of the challenges, two years after project start, is to maintain the ethics reflection groups, particularly if the facilitators quit their job. A plan for training new facilitators is thus important.

Another barrier is lack of time and thus problems with prioritising participation. While some might consider it an expense, the participants evaluate ethics reflection groups to be an investment. Taking the time to stop and think, are considered to be a good investment*.* This is a way of thinking that we have learned requires a manager who supports the measure fully. Managers’, especially ward managers’ support and commitment are essential for success. Therefore, we recommend postponing the initiation if it is not possible to involve the managers.

Initially, we wanted the reflection groups to be multidisciplinary, something that we only partially succeeded in. For example, it was very rare that doctors or physiotherapists participated. Despite the limited data, we have positive experiences that suggest that multidisciplinary groups have a positive impact on the reflection process.

### Methodological limitations

Our research has limitations. More research on this topic (including other services, more and other types of participants, e.g. patients/proxies and more rigorous study design) is needed to assess whether our positive findings are representative. The strength of our study is that we have different groups of informants (employees, facilitators, managers) in addition to observations and written notes. Other strengths are that we as researchers have been closely involved in the project and therefore have detailed and firsthand knowledge about the intervention and the implementation process. But our closeness and involvement in the project could conceivably have influenced the findings, and is thus also a weakness of the study.

The participants in these interviews are all professionals, either responsible for managing or providing health care. To the extent we know anything about what patients, residents and their families think about these questions, we know it just indirectly, through the managers’ and health care professionals’ assumptions and views. Those who have received the service may consider it differently.

## Conclusions

The managers, facilitators and staff participating in this study experienced ethics reflection groups as very valuable. The time and resources needed were considered to be highly rewarding investments by all the participants. Another major finding was the importance of management support and anchoring ethics sessions in the routine of daily work. Ethics reflection groups, as implemented in this study, may lead to collegial support and cooperation, improved practice, better involvement of patients and their families, personal and professional development and mutual learning among employees.

Several aspects seem to be important for the positive experience: The reflection process focusing on the participants’ own ethical challenges from their daily work; and the reflection process taking place in the employee’s workplace and being led by a colleague trained in facilitation.
